# Coordinate value of the femoral head center estimated using those of the tip of the greater trochanter and lesser trochanter

**DOI:** 10.1038/s41598-023-30063-7

**Published:** 2023-02-16

**Authors:** Norio Imai, Keishi Kimura, Atsushi Sakagami, Asami Nozaki, Yoji Horigome, Hayato Suzuki

**Affiliations:** 1 Division of Comprehensive Musculoskeletal Medicine, Niigata University Graduate School of Medical and Dental Sciences, 1-757, Asahimachi-Dori, Chuo Ward, Niigata, 9518510 Japan; 2 Division of Orthopedic Surgery, Department of Regenerative and Transplant Medicine, Niigata University Graduate School of Medical and Dental Sciences, Niigata, Japan

**Keywords:** Diseases, Medical research

## Abstract

Several studies have reported estimating the femoral head center (FC) from reference points on the pelvis; however, none have reported estimates obtained from those on the femur. In this cross-sectional study, we investigated the estimated point of FC from the coordinate value of the tip of the greater trochanter (GT) and lesser trochanter (LT) using a formula with a three-dimensional measurement technique. We used data from 92 healthy Japanese subjects without any back or knee symptoms and no abnormalities in the hip, knee, or spine on plain radiographs. In our study, the difference in the anteroposterior direction was larger than that in the other directions. We speculate that the accuracy of defining the tip of the LT is difficult in the anteroposterior direction. Moreover, the correlation coefficients were larger for women. The reason for this was unclear because the variation in the proximal femur may be similar in women. We found that the average difference between the actual and calculated values was approximately 2 mm. We considered that the coordinate value of the FC from the tip of the GT could be estimated more accurately using the regression equation compared to previous methods based on pelvic reference points.

## Introduction

Previously, the adjustment of femoral offset, global femoral offset (the sum of femoral offset), and acetabular offset are considered essential in total hip arthroplasty^1^. A suitable offset contributes to a decrease in polyethylene liner wear^2,3^, preservation of the power of the abductor muscles around the hip joint^2^, maintenance of hip-joint range of motion^4^, and function of the hip joint^1,4^. However, it is not always easy to detect the original femoral head center (FC), such as in patients with bilateral hip osteoarthritis with severe arthritic changes. Therefore, FC should be estimated using other reference points on the femur.

Several studies have reported the estimation of FC from the pelvic bone using the point of the anterior superior iliac spine and obturator foramen via three-dimensional methods^5,6^ and the point of the anterior superior iliac spine and pubic symphysis in two-dimensional^7,8^ and three-dimensional analysis^9,10^. However, there are no reports on estimated FC from the reference point of the femur.

Several studies reported that the orientation of the lesser trochanter (LT) was strongly and linearly related to femoral neck anteversion^11–13^; therefore, we hypothesized that the shape of the proximal femur above the LT was similar, and the point of the femoral head could be estimated from the reference points in the proximal femur, such as the tip of the greater trochanter (GT) and LT. We aimed to investigate the estimated point of FC from the coordinate value of the tips of the GT and LT using a formula with a three-dimensional measurement technique.

## Methods

For this cross-sectional study, we used data of 184 femurs from 92 healthy Japanese subjects (50 men, average height 168.8 ± 6.5 cm, average age of 46.6 ± 20.2 years; 42 women, average height 156.6 ± 6.9 cm, average age of 48.6 ± 18.5 years), who were family members of outpatients and medical staff at our hospital. The study included data collected from January 1, 2010, to December 31, 2012. The study details were displayed in the outpatient clinic of the orthopedic surgery department to inform patients. The target measurement was a morphological analysis of the spatial alignment between the (1) femur and tibia, and (2) pelvis, hip, and knee in normal subjects. Data on the subjects were described regarding the spatial alignment of the femur and tibia from a previous study^14–16^ and were used with written permission. The participants had no back or knee symptoms.

No abnormalities were observed on the plain radiographs of the hip, knee, or spine. CT was performed on all subjects to reconstruct the 3D bone models of the pelvis and femur, as described previously^14–16^. A multislice CT scanner with a 64-row detector (Aquilion64™; Toshiba Medical Systems, Otawara, Tochigi, Japan) acquired approximately 500 slices (slice thickness, 1.25 mm) from each participant. The examined area consisted of the most proximal part of the pelvis and the most distal part of the femur. The scan parameters were 120 kV for tube voltage; 100 mA, tube current; 14.60 mGy, CT dose index volume; and 896.7 ± 129.9 mGy·cm, mean dose-length product. These parameters were used in a previous study^17^. The Institutional Review Board of Niigata University approved the study design (approval no. 2020-0137) and also waived the requirement of obtaining informed consent owing to the cross-sectional, retrospective, and non-interventional nature of the study. All the methods in this study were performed according to relevant guidelines and regulations.

**Figure 1:**
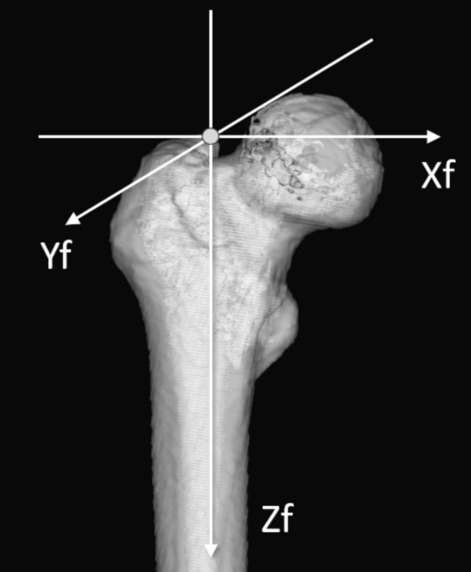
The coordinate system of the femur.

The 3D model of the femur faced the retrocondylar plane, including the most posterior point of the bilateral posterior condyles and the most posterior point of the GT^18^, as the coordinate system of the femur. The femoral axis was defined as a line connecting the knee center and trochanteric fossa. In the femoral coordinate system, the unit vector was defined as follows: the Zf axis was the line where the femoral axis was projected onto the retrocondylar plane; the Xf axis was at the right angle to the Xf axis on the retrocondylar plane; and the Yf axis was at the right angle to the Xf and Zf axes, similar to our previous studies^19,20^ (Fig. 1).

**Figure 2:**
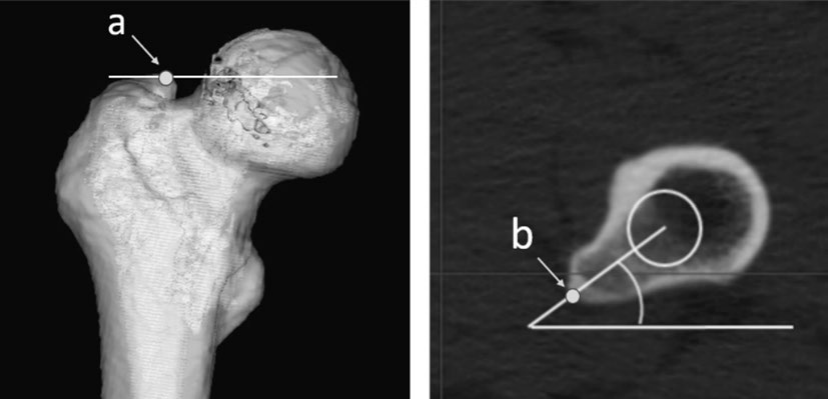
The definition of the tips of the greater and lesser trochanters. (**a**) Tip of the greater trochanter; (**b**) the tip of the lesser trochanter was defined as in the study by Unlu et al.^13^.

**Figure 3:**
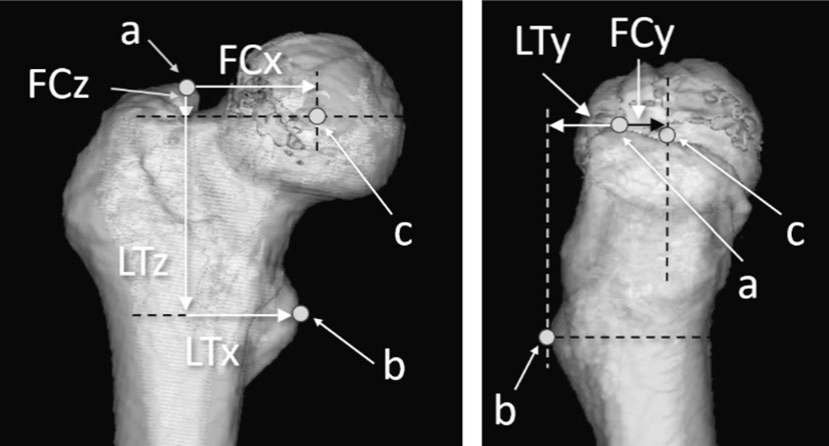
The measurement of the length from the tip of the greater trochanter and the lesser trochanter. (**a**) Tip of the greater trochanter, origin point for measurement; (**b**) tip of the lesser trochanter; (**c**) femoral head center.

The tip of the GT was defined as the most proximal point on the plane containing the Xf and Zf axes. According to a previous study, the tip of the LT was defined as the most prominent point on the plane containing the Xf and Zf axes^13^. The length between the tip of the GT and FC was defined as FCx on the Xf axis, FCy on the Yf axis, and FCz on the Zf axis (Fig. 2). Similarly, the length between the tip of the GT and the tip of the LT was defined as LTx on the Xf axis, LTy on the Yf axis, and LTz on the Zf axis (Fig. 3). The directions were expressed in plus values from the tip of the GT, medially in the Xf axis, anteriorly in the Yf axis, and distally in the Zf axis.

We calculated the coordinate value from the tip of the GT relative to FC in each Xf, Yf, Zf direction, FCx, FCy, and FCz, respectively, from the coordinate values of the tip of the LT and the tip of the GT relative to the FC using multiple regression analysis. Intrarater and interrater reliabilities were evaluated for each parameter using intraclass correlation coefficients (ICCs). Intrarater reliability was evaluated by having the same observer repeat the measurements at intervals of at least 1 week. Interrater reliability was evaluated by having an additional observer repeat the measurements, as performed in our previous study^17^. We evaluated all of the statistical analyses using SPSS Ver. 28 (SPSS, Inc., Chicago, IL). Concerning the multiple regression analysis and ICCs, we considered a two-tailed *p*-value of < 0.05 as statistically significant. Regarding the correlation analysis, we used a post hoc analysis to evaluate the statistical power (type II (β) error), with 0.3 as effect size (d) of 0.05, and a type I (α) error.

## Results

Details of the participants are presented in Table 1. FCz was positive in men and negative in women; consequently, FC was placed above the tip of the GT in men and below in women (Table 2). The correlation coefficients ranged from 0.515 to 0.911 (Table 3), and the difference between the actual and calculated values was approximately 2 mm (1.16 to 3.48 mm) on average (Table 4).

**Table 1:** The details of the subjects.

	Men	Women
Age (years)	47.3 ± 18.4	49.1 ± 17.2
Body height (cm)	169.0 ± 6.5	156.4 ± 6.8
Body weight (Kg)	63.3 ± 8.3	52.4 ± 7.3

All values are expressed as mean ± standard deviation.

**Table 2:** The length from the tip of the greater trochanter to the femoral head center and lesser trochanter.

	Men	Women
FCx (mm)	45.3 ± 4.7	38.8 ± 4.4
FCy (mm)	18.7 ± 7.1	21.0 ± 7.3
FCz (mm)	6.8 ± 4.2	− 6.5 ± 4.7
LTx (mm)	36.8 ± 4.7	34.0 ± 5.9
LTy (mm)	− 5.9 ± 6.9	− 2.2 ± 7.5
LTz (mm)	62.2 ± 4.5	42.8 ± 8.9

All values are expressed as mean ± standard deviation.

**Table 3:** The formulae calculated with the multiple regression analysis.

	Formulae	Correlation coefficient
Men	FCx = 0.504 × LT_X_ − 0.475 × LT_Y_ + 23.755	0.682*
	FCy = 0.715 × LT_Y_ + 22.416	0.672*
	FCz = − 0.331 × LT_X_ − 0.285 × LT_Z_ + 1.451	0.515*
Women	FCx = 0.331 × LT_X_ − 0.499 × LT_Y_ + 26.529	0.787*
	FCy = 0.811 × LT_Y_ + 22.793	0.833*
	FCz = − 0.477 × LT_Z_ − 26.915	0.911*

*p < 0.01.

**Table 4:** The differences between the measured and calculated values.

	Men	Women
FCx (mm)	2.41 ± 1.85	2.17 ± 1.64
FCy (mm)	3.78 ± 2.76	3.11 ± 2.56
FCz (mm)	2.81 ± 2.20	1.53 ± 1.16

All values are expressed as mean ± standard deviation.

**Figure 4:**
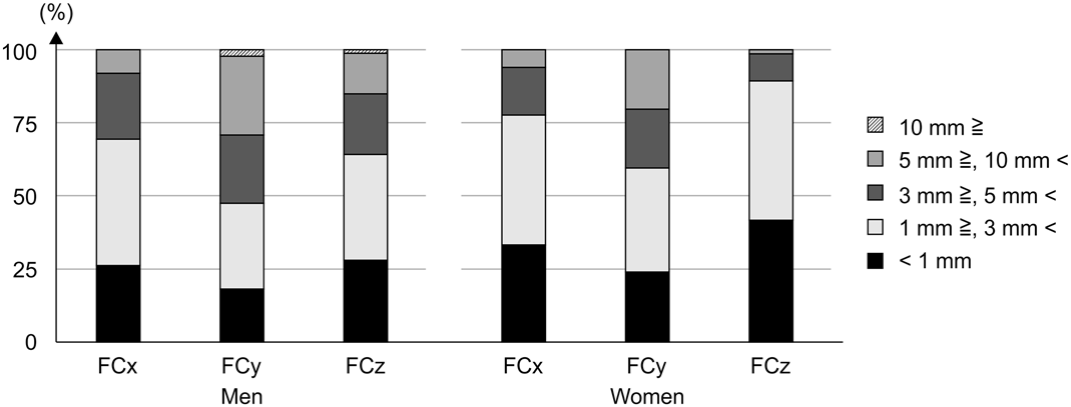
The differences between actual and estimated values. The differences were within 5 mm in FCx and FCz among both men and women.

We estimated 47 to 69% and 59.5 to 89.3% of each length within 3 mm, and 71 to 91% and 79.8 to 98.8% of each length within 5 mm in men and women, respectively (Fig. 4). The power analysis of the correlation showed power values of 0.905 and 0.813 for men and women, respectively. We obtained ICCs > 0.9 for all measurements, indicating high intraobserver and interobserver reliability (Table 5).

**Table 5:** Intrarater and interrater reliabilities.

	Intrarater		Interrater	
	MAD (mm)	ICC	MAD (mm)	ICC
FCx	0.55 ± 0.98	0.961*	0.70 ± 1.37	0.933*
FCy	0.62 ± 1.50	0.94 l*	1.06 ± 1.51	0.911*
FCz	0.57 ± 0.75	0.969*	0.80 ± 1.06	0.944*
LTx	1.19 ± 1.11	0.934*	1.20 ± 1.56	0.919*
LTy	1.22 ± 1.95	0.894*	2.00 ± 2.05	0.833*
LTz	1.85 ± 1.53	0.925*	1.95 ± 1.92	0.875*

*p < 0.01.

## Discussion

This study found that the average difference between the actual and calculated values was approximately 2 mm. We assumed that the coordinate value of the FC from the tip of the GT could be estimated using a regression equation. Several studies have described the estimation of FC using two-dimensional procedures^7,8,21,22^. However, two-dimensional analysis with regard to the anteroposterior dimension could not be performed. These two-dimensional methods were very practical; however, if the surgeons would like to restore the femoral head center, determining the anteroposterior offset using three-dimensional method seems essential, because two-dimensional methods do not consider the decrease of femoral offset when stem anteversion is increased. Moreover, two-dimensional methods may be greatly affected by the position, especially the rotation of the femur. Therefore, we believe this three-dimensional estimation is valuable. In planning THA, if the hip center is located medially due to the insertion of the acetabular component, the distance of translation of the hip center should be added to the estimated value of FCx.

Several studies have estimated the coordinate value of the FC from reference points on the pelvis, such as the anterior superior pelvic plane, pubic symphysis, and obturator foramen^5,6,9,10^. Based on these results, the accuracy of the estimation was approximately 3–5 mm. Therefore, our method was considered to estimate the values by using the reference points of the femur, and we considered our paper to be the first report on this method which is more accurate than previous methods that calculated the coordinate value of the FC with pelvis reference points, which is a strong point in this study.

In our study, the difference in FCy was greater than that in FCx and FCz. We speculate that the accuracy of defining the tip of the LT was challenging in the anteroposterior direction. However, we previously found that it is essential to maintain global femoral offset in the X-direction in this study and leg length discrepancy in the Z-direction in this study to improve clinical outcomes^23^; therefore, this may not have considerable importance practically. Moreover, we found that the correlation coefficients were larger for women than for men. The reason for this was unclear in our analysis because the variation in the proximal femur may be similar in women.

This study has several limitations. First, only a small number of participants were included, and only Japanese individuals were enrolled. However, the power of the analysis was greater than 0.8. Second, this study only included healthy subjects. For this reason, while our findings can be applied to patients with osteonecrosis of the femoral head or femoral neck fracture, it is unclear whether they should be applied to patients with hip dysplasia. Patients with hip dysplasia should be examined further in the future. Finally, our method using the tips of the greater and lesser trochanters may be considered somewhat complicated. We aim to identify simpler and faster estimation methods for the clinical setting.

## Conclusion

We found that the average difference between the actual and calculated values was approximately 2 mm. We considered that the coordinate value of the FC from the tip of the GT could be estimated using the regression equation more accurately than previous methods that calculated the value using the pelvic reference points.

## Data Availability

The data that support the findings of this study are available from the corresponding author, NI, upon reasonable request.
